# Mechanical Property Modulation Mechanisms and Strengthening Strategies for Lightweight Thermal Insulation Refractories: A Review

**DOI:** 10.3390/ma19194062

**Published:** 2026-09-23

**Authors:** Silin Chen, Jingjing Liu, Gang Wang, Bo Ren

**Affiliations:** 1 Technical Support Center for Prevention and Control of Disastrous Accidents in Metal Smelting, University of Science and Technology Beijing, Beijing 100083, China; 2 School of Materials Science and Engineering, University of Science and Technology Beijing, Beijing 100083, China

**Keywords:** lightweight thermal insulation refractories, degradation mechanism, mechanical properties, pore structure, strengthening strategies

## Abstract

Lightweight thermal insulation refractories (LTIRs) face an inherent trade-off between thermal insulation and mechanical integrity under harsh, multi-physical service environments. However, existing research largely focuses on isolated strengthening techniques and lacks an integrated understanding of multi-field coupled progressive degradation and synergistic design principles. Addressing this gap, this review establishes an integrative framework linking damage physics to full-chain strengthening strategies. We first delineate a three-stage progressive damage model under coupled thermo-mechano-chemical fields, spanning microcrack initiation, corrosion-induced volume distortion, and creep-driven skeletal collapse. Subsequently, reinforcement breakthroughs are systematically evaluated across four complementary dimensions: pore micro-architecture engineering, pore-wall densification alongside bonding-phase reinforcement, multi-scale toughening, and heterogeneous interface engineering. Finally, to resolve intrinsic performance conflicts, we deliver actionable guidelines and a service-condition-based design map that synergizes pore spheroidization, in situ crystalline interlocking, and dynamic interfacial stress relaxation. This work provides theoretical guidance and a practical roadmap for the rational design of highly damage-tolerant, energy-efficient refractories tailored for extreme industrial conditions.

## 1. Introduction

Lightweight thermal insulation refractories (LTIRs) are indispensable materials for high-temperature industrial processes such as metallurgy, cement manufacturing, and power generation, as well as aerospace thermal protection systems, owing to their superior thermal barrier performance and ultra-low thermal conductivity [1,2]. Driven by global decarbonization goals and increasingly stringent service conditions, improving their long-term comprehensive performance has become urgent. Nevertheless, the high porosity required to achieve low thermal conductivity inevitably disrupts the continuity of the solid matrix, which leads to substantial deterioration in mechanical properties, including room and high-temperature strength, thermal shock resistance (TSR), and high-temperature creep resistance [3]. Such property degradation further undermines service reliability under practical coupled thermo-mechano-chemical environments. Therefore, maintaining improved mechanical integrity while retaining high porosity remains a major bottleneck and long-standing challenge in advanced refractory materials engineering.

To mitigate this mechanical performance degradation, extensive efforts have been devoted to pore architecture tailoring, compositional and sintering optimization, second-phase reinforcement, and interface engineering [4,5]. In terms of pore structure regulation, constructing closed pores, refining micro/nanoscale pores, and designing hierarchical pore architectures in LTIRs can effectively relieve stress concentration and hinder crack propagation without sacrificing high porosity [6,7]. This review critically distinguishes the characteristics of isolated closed pores, interconnected open pores, and hierarchical bimodal/multimodal pore networks. Even at identical total porosity, these distinct pore topologies produce drastically different mechanical, thermal, and damage-tolerant behaviors. Since the thermal and mechanical performances of LTIRs are strongly dictated by chemical composition and pore structure, this review systematically analyzes the mechanical evolution mechanisms of five representative LTIR systems: silica-based, cordierite/anorthite-based, mullite-based, alumina-based, and zirconia/high-entropy ceramic refractories.

For compositional and sintering optimization, liquid-phase sintering enabled by sintering aids and the in situ generation of bonding phases can densify pore walls and thereby enhance the overall structural integrity [8]. As for second-phase modification, transformation toughening (e.g., ZrO_2_), together with whisker/fiber bridging, pull-out and crack deflection, has been verified to dissipate fracture energy in various ceramic systems [9,10,11]. Moreover, interface engineering is another powerful strategy to enhance TSR by introducing gradient transition layers and rationally designed weak interfaces [12,13]. Despite these substantial advances, two critical research gaps still exist. First, conventional static degradation models cannot describe dynamic damage evolution under multi-field coupling during service, making quantitative characterization of microdamage progression difficult. Second, most existing studies adopt separate strengthening strategies rather than an integrated design combining pore architecture, pore-wall densification, multi-scale reinforcement, and heterogeneous interfaces, which limits the guidance for multi-objective performance optimization of LTIRs.

Addressing the strength–insulation trade-off and the need for enhanced damage tolerance under extreme service, this review summarizes recent advances in mechanical modulation and toughening of LTIRs. Focusing on oxide LTIRs for thermal insulation above 800 °C, five systems governed by distinct chemical bonding and crystalline phases are evaluated: silica-based (nanoporous; ultra-low conductivity), cordierite/anorthite-based (ultra-low expansion; high TSR), mullite-based (interlocking needles; creep resistance), alumina-based (corundum matrix; high load-bearing), and zirconia/high-entropy ceramic-based (transformation toughening; extreme-temperature stability). Using fracture mechanics and cellular solid theories, we establish a three-stage progressive damage model under coupled fields and then critically examine strengthening strategies across four dimensions: pore structure design, pore-wall densification via in situ bonding phases, multi-scale reinforcement, and heterogeneous interface engineering. On this basis, we dissect the performance conflicts of isolated strengthening routes and propose a multi-scale synergistic design methodology. Finally, key bottlenecks and future directions are highlighted, establishing a theoretical framework for the customized design of lightweight and high-strength refractories.

## 2. Review Methodology and Contribution Framework

To ensure a systematic and reproducible coverage of mechanical property modulation and strengthening strategies for LTIRs, we conducted a comprehensive literature search across Web of Science, Scopus, and Google Scholar for the period from January 2000 to June 2026. The search employed combinations of keywords including “lightweight thermal insulation refractories”, “porous insulating ceramics”, “mechanical properties”, “strengthening strategies”, “pore structure”, “thermal shock resistance”, “toughening mechanisms”, and “interface engineering”. Only peer-reviewed experimental or theoretical studies that provided quantitative data on porosity, mechanical strength, thermal conductivity, or TSR were included; studies on non-refractory or non-ceramic materials (e.g., polymer foams, organic aerogels) and those lacking sufficient experimental detail were excluded. From the retrieved literature, 90 key references were selected for critical analysis.

The selected studies were categorized into four thematic strengthening dimensions, (i) pore structure design and regulation, (ii) pore-wall densification and in situ bonding phase formation, (iii) multi-scale reinforcement toughening, and (iv) heterogeneous interface engineering, which form the organizational backbone. This classification not only reflects the prevailing research landscape but also enables a systematic dissection of the distinct physical mechanisms and performance trade-offs inherent to each strategy.

As summarized in Table 1, previous reviews have individually addressed aspects such as porous ceramic processing, thermal insulation performance, or the TSR of dense ceramics. However, none has established an integrated framework that systematically links the multi-field coupled degradation mechanisms (thermo-mechano-chemical) of LTIRs to a comprehensive portfolio of strengthening strategies, spanning pore architecture, pore-wall densification, multi-scale reinforcement, and interface engineering. The present review fills this gap by (1) proposing a three-stage progressive damage model under coupled service fields, (2) critically evaluating strengthening strategies across four complementary dimensions with quantitative comparative analysis, (3) identifying synergistic effects and performance conflicts, and (4) providing a service-condition-based design map for rational strategy selection. These elements collectively constitute the primary contributions of this work, distinguishing it from the prior literature.

## 3. Mechanical Damage Mechanisms and Enhancement Bottlenecks of LTIRs During Service

### 3.1. Degradation Mechanisms Under Harsh Service Environments

In industrial thermal equipment, LTIRs are continuously subjected to severe coupled thermo-mechano-chemical environments that vary markedly across service scenarios [17]. Thermal cycling typically imposes macroscopic temperature gradients of 200~1200 °C with furnace heating/cooling rates of 10~50 °C·min^−1^, whereas extreme operational transients (such as emergency shutdowns or direct liquid quenching) can impose localized cooling rates that exceed 200 °C·s^−1^. Under mechanical loading, sustained structural self-weight and operational constraints induce compressive creep stresses typically ranging from 0.1 to 2.0 MPa at 1200~1600 °C. Concurrently, aggressive chemical species—including alkali metal vapors (e.g., K_2_O, Na_2_O) at 900~1400 °C, molten silicate slags at 1000~1600 °C, and combustion atmospheres containing reactive O_2_, CO, and H_2_O steam—rapidly invade the interior.

Under such multiphysical coupling, the macroscopic degradation of LTIRs is fundamentally driven by a three-stage progressive damage process, namely, thermally induced microcracking (Stage I), chemically driven phase evolution and volumetric distortion (Stage II), and creep-mediated skeletal collapse (Stage III), as schematically depicted in Figure 1.

#### 3.1.1. Stage I: Thermo-Mechanically Induced Microcracking and Stress Concentration at Pore Walls

During rapid thermal transients, steep temperature gradients generate severe localized thermal stresses within the cellular skeleton, which are intensified by mismatches in the thermal expansion coefficient (TEC, Δα) and elastic modulus between constituent phases [18]. Because porous ceramics possess a reduced solid load-bearing cross-section, localized stresses are significantly magnified at geometric discontinuities—such as sharp pore corners, thin struts, and grain boundaries. Once the local transient tensile stress exceeds the intrinsic fracture strength of the pore walls, microcracks preferentially nucleate at these stress-concentrating sites. Under cyclic thermal loading, these nascent microcracks propagate, branch, and coalesce along pore perimeters and inter-pore struts, precipitating localized spalling and a progressive decline in effective load-bearing capability [19].

The susceptibility to crack initiation is intrinsically governed by the thermophysical properties of the refractory matrix:

(1) Low-CTE matrices: Cordierite-based systems (Δα ≈ 2.0 × 10^−6^ K^−1^) generate thermal stresses typically below 20 MPa even under a temperature shock of ΔT = 1000 °C and exhibit superior intrinsic resistance to crack nucleation.

(2) High-CTE matrices: Alumina-based systems (Δα ≈ 8.5 × 10^−6^ K^−1^) develop transient tensile stresses exceeding 150 MPa at pore boundaries during rapid quenching (≥50 °C·s^−1^), inevitably leading to the precipitation of premature microcrack networks.

Furthermore, the resultant residual stress field—composed of tensile driving regions and compensatory compressive zones—continually alters the effective elastic modulus and fracture toughness. In ZrO_2_-containing systems, the phase transformation (t → m) induces localized compressive residual stress that blunts propagating crack tips [20]. In contrast, in multi-phase alumina- or mullite-based composites, thermal expansion mismatches between the primary matrix and secondary phases frequently establish harmful localized tensile residual stresses, which serve as pre-existing driving forces for thermal shock cracking.

Quantifying these micro-scale stress fields in porous cellular architectures remains an experimental bottleneck. While X-ray diffraction (sin^2^ψ method) is restricted to near-surface layers and suffers from severe scattering over porous surfaces, neutron diffraction offers non-destructive bulk mapping but requires large-scale facilities [21]. Concurrently, digital image correlation (DIC) combined with finite element (FE) modeling provides dynamic full-field surface strain fields under load, yet direct in situ quantification of initial fabrication-induced residual stresses within high-porosity skeletons remains scarce.

#### 3.1.2. Stage II: Chemically Driven Phase Evolution and Volumetric Distortion

The microcrack networks formed in Stage I, together with intrinsically interconnected open pores, serve as fast diffusion channels for aggressive gaseous and liquid media [22]. Chemical attack at elevated temperatures drastically destabilizes the phase constitution and structural stability of LTIR skeletons via two primary pathways:

(1) Fluxing and liquid-phase formation: In oxide systems (e.g., silica-, cordierite-, and mullite-based LTIRs), infiltrated alkali vapors (K_2_O/Na_2_O) or molten slags react with the aluminosilicate skeletons above 1000 °C, depressing the eutectic solidus temperature by 200~400 °C. This triggers the premature generation of low-viscosity feldspathic and nepheline liquid melts, which accelerate dissolution-precipitation, thin the solid struts, and severely degrade the high-temperature load-bearing capacity.

(2) Volumetric dilation and hoop stresses: In non-oxide or non-stoichiometric high-entropy systems (e.g., porous high-entropy carbides and nitrides), in situ oxidation yields secondary oxide scales (such as SiO_2_ or complex rutile-type phases) accompanied by a 5~15% molar volume dilation. This anisotropic volumetric expansion generates high secondary circumferential tensile stresses (often reaching 50~200 MPa) around the pore walls, which drives secondary crack multiplication and macroscopic structural spallation [23].

#### 3.1.3. Stage III: Creep-Mediated Structural Degradation and Skeletal Collapse

During prolonged high-temperature exposure under sustained mechanical constraints, LTIRs enter the final stage of degradation, governed by coupled high-temperature creep and microstructural coarsening. Under sustained compressive stresses of 0.1~2.0 MPa at 1200~1600 °C, cellular ceramic frameworks exhibit macroscopic creep strain rates on the order of 10^−5^~10^−3^ h^−1^, primarily driven by grain-boundary sliding, diffusion-controlled mass transport (Coble and Nabarro-Herring creep), and viscous flow of intergranular glassy films.

Simultaneously, the high specific surface energy of porous materials drives aggressive Ostwald ripening and sintering shrinkage. Small grains coarsen (typically expanding from 1~5 μm to 10~50 μm during extended campaigns), promoting pore coalescence, cell-strut thinning, and eventual structural buckling [24]. This skeletal densification and pore collapse not only lead to an irreversible loss of geometric stability but also drastically reduce phonon/photon scattering boundaries, thereby causing catastrophic degradation of both thermal barrier efficacy and structural reliability.

Correspondingly, Table 2 systematically compiles the dominant degradation mechanisms, critical triggering operational boundaries (e.g., temperature gradients, quenching rates, and atmospheric compositions), and recommended diagnostic methodologies for each stage. By aligning multi-scale experimental tools—spanning in situ high-temperature mechanical probing and real-time strain tracking (DIC, acoustic emission), three-dimensional morphological tracking (micro-CT), and post mortem chemical spectroscopy (SEM-EDS, Raman/FTIRs)—with specific damage stages, this framework provides both a practical guide for industrial failure diagnosis and a quantitative benchmark for calibrating the dynamic multi-field damage models discussed below [25].

### 3.2. Theoretical Bottlenecks in Mechanical Performance Enhancement

#### 3.2.1. Classical Empirical Scaling Laws and Their Intrinsic Constraints

The fundamental mechanical bottleneck of LTIRs stems from their high porosity: as void volume expands to cut off thermal conduction, the solid load-bearing cross-section is drastically reduced, and stress concentrations around pore boundaries escalate sharply. In classical ceramic mechanics and cellular solid physics, this strength–porosity trade-off has traditionally been interpreted through three core analytical frameworks spanning macroscopic, mesoscopic, and microscopic scales:

(1) Macro-scale solid skeleton reduction:

At the macro scale, the degradation of load-bearing capacity with total porosity (*P*) is widely described by the empirical Ryshkevitch–Duckworth relation (Equation (1)) [26]:

(1) $$\sigma =\sigma_{0}\exp(-BP)$$

where *σ* and *σ*_0_ represent the strengths of the porous and fully dense materials, respectively; *P* is the total porosity; and *B* is the structural empirical constant closely governed by geometric features such as pore size, aspect ratio, and orientation. Typical values of *B* for LTIRs range from 2 to 7, with higher values indicating stronger strength degradation per unit porosity increase.

Additionally, for cellular networks featuring distinct cell struts and open cavities, the classical Gibson–Ashby model expresses the relative elastic modulus (*E/E*_0_) as a power-law function of relative density [27]:

(2) $$\frac{E}{E_{0}}=C{\left(\frac{\rho }{\rho_{0}}\right)}^{n}=C{\left(1-P\right)}^{n}$$

where *E* and *E_0_* are the elastic moduli of the porous and fully dense skeletons, respectively; *ρ/ρ*_0_ is the relative bulk density; *C* is a structural geometric constant (typically *C*~1); and the exponent *n* reflects the cell deformation mode (e.g., *n* = 2 for bending-dominated struts in open-cell foams, and 1 < *n* < 2 for closed-cell foams with membrane-stretching behavior). While Equation (1) provides superior fitting flexibility for sintered refractories containing irregular, isolated pores, Equation (2) offers a deterministic physical description for open-celled cellular architectures.

(2) Microscale stress amplification and defect-governed fracture:

At the micro scale, pores act not merely as missing mass but as critical stress concentrators. According to the modified Griffith–Irwin brittle fracture criterion, the critical fracture stress *σ_f_* is dictated by the characteristic dimension of the fatal internal defect [28]:

(3) $$\sigma_{f}=\sqrt{\frac{2E\gamma_{s}}{\pi \alpha }}$$

where *σ_f_* is the critical fracture stress, *E* is the elastic modulus, *γ_s_* is the fracture surface energy, and *α* represents the characteristic dimension of internal defects. In LTIRs, large interconnected pores or angular pore corners serve as intrinsic flaw origins $\sigma_{f}\propto d_{pore}^{-1/2}$, forcing external stress trajectories to funnel through narrow cell struts and precipitate premature crack nucleation at stress levels far below theoretical matrix strength.

(3) Mesoscopic degradation in crack propagation resistance:

Void spaces also severely curtail the energy-dissipating pathways available ahead of advancing crack fronts. The effective fracture toughness (*K_IC_*) decays exponentially with porosity following the Ryshkevitch-type empirical scaling law [29], an extension of the well-known Ryshkevitch–Duckworth strength relation for porous ceramics:

(4) $$K_{IC}=K_{0}\exp(-bP)$$

where *K_IC_* stands for the fracture toughness, *K*_0_ denotes the fracture toughness of the dense counterpart, *P* is the total porosity, and *b* is a structural constant typically ranging from 2 to 6, heavily dependent on pore size and shape. When pores exhibit irregular polyhedral morphologies or sharp angular corners, stress concentration effects become remarkably pronounced. Under applied external loads, these sites readily trigger atomic bond rupture and microcrack nucleation, ultimately leading to insufficient fracture toughness.

(4) Inherent breakdown of classical models in LTIR service:

Although Equations (1)–(4) establish the foundational baseline for porous ceramics, they suffer from critical intrinsic limitations when applied to practical LTIRs under harsh thermal equipment conditions:

Scalar oversimplification of pore topology: Classical models treat porosity *P* as an isotropic, single-valued scalar parameter. They completely overlook spatial connectivity (isolated closed pores vs. open channels), pore sphericity, and multimodal pore-size distributions (e.g., bimodal hierarchies). As demonstrated in Section 4.1, porous architectures with identical total porosity can exhibit disparities exceeding 300% in strength and an order of magnitude in thermal conductivity depending on whether pores are closed spheres or tortuous interconnected networks.

Static assumption vs. dynamic service degradation: Classical formulations assume chemically inert, time-invariant, and isothermal microstructures. In actual thermo-mechano-chemical environments (Section 3.1), thermal shock cycling generates transient residual stress fields; alkali-slag corrosion erodes pore-wall thicknesses; and high-temperature creep induces continuous pore buckling (Figure 1). Consequently, static scaling laws cannot capture the progressive decay of effective modulus (*E*_t_), yield strength (*σ_t_*), and damage tolerance during prolonged campaigns [30].

#### 3.2.2. Multi-Field Coupled Modeling Frameworks: Current Advances and Persistent Challenges

To move beyond static empirical scaling laws, advanced numerical and analytical modeling frameworks have been developed to capture multiphysical damage evolution in porous refractories. As categorized in Table 2, current modeling strategies encompass four main methodologies:

(1) Fracture-mechanics-based crack propagation models: These incorporate stress intensity factors and cohesive zone formulations to track crack paths along pore struts. However, they struggle to capture dynamic creep relaxation and phase-corrosion interactions at elevated temperatures.

(2) Continuum damage mechanics (CDM): By defining anisotropic internal damage tensors (D*_ij_*), CDM effectively calculates progressive macroscopic stiffness loss and thermal fatigue degradation. Nonetheless, CDM models rely heavily on phenomenological parameters, which are extremely difficult to calibrate for heterogeneous cellular refractories at temperatures above 1200 °C.

(3) Coupled thermo-mechanical representative volume element (RVE) simulations: By reconstructing realistic three-dimensional (3D) cell skeletons via micro-CT, RVE-based finite element methods directly evaluate transient thermal stress peaks and localized plastic strains [31]. Nevertheless, tracking microstructural evolution (e.g., grain growth, Ostwald ripening of pores, and melt dissolution) within multi-scale RVEs imposes an intractable computational burden.

(4) Phase-field modeling (PFM) of multi-physics fracture: PFM has emerged as a powerful tool for predicting complex crack branching and coalescence without ad hoc tracking algorithms. However, integrating chemical diffusion potentials (e.g., alkali melt invasion) into thermo-mechanical PFM formulations remains in its infancy.

In summary, quantitative life-cycle damage prediction for LTIRs remains fundamentally constrained by three interrelated bottlenecks: (i) the difficulty of decoupling the concurrent, strongly synergistic interactions among thermal fatigue, high-temperature creep, and chemical corrosion, which cannot be reduced to simple linear superposition; (ii) the continuous service-induced evolution of pore connectivity, strut aspect ratios, and interfacial phases, which renders static initial-state parameters obsolete; and (iii) the scarcity of high-fidelity calibration data—such as real-time full-field strains, localized creep kinetics, and high-temperature chemical diffusion rates—required for rigorous empirical validation. Addressing these theoretical and computational challenges therefore demands a shift beyond purely predictive modeling toward the multi-dimensional strengthening strategies and service-tailored design roadmaps developed in the subsequent sections.

## 4. Strategies for Enhancing the Mechanical Properties of LTIRs

As illustrated in Figure 2 and contextualized with the intrinsic thermophysical properties of typical LTIR systems, the mechanical property enhancement strategies are categorized into four complementary and hierarchically integrated dimensions: (1) optimizing pore structure design to alleviate thermal stress concentration and suppress microcrack initiation at the source; (2) densifying pore walls and reinforcing bonding phases to hinder the diffusion of corrosive species while enhancing the chemical stability of the matrix; (3) introducing multi-scale reinforcements; and (4) employing heterointerface engineering to combat high-temperature creep and skeleton collapse, thereby imparting the porous architecture with durable deformation resistance and self-healing capability. Accordingly, a full-chain strengthening strategy integrating damage inhibition, diffusion pathway blockade, and structural anti-instability is constructed.

### 4.1. Pore Structure Design and Regulation

As the main constituent of LTIRs, pores significantly govern the mechanical properties of the material by adjusting their structural parameters. Elevated porosity diminishes the solid area per unit volume and leads to strength degradation. Irregular pores easily trigger stress concentrations and become sources of microcrack initiation. Therefore, optimizing pore structure is essential to enhance the mechanical performance of LTIRs, with pore morphology and size as primary optimization targets.

#### 4.1.1. Design of Closed Pores and Pore Spheroidization

Compared with open pores, closed pores can effectively mitigate crack-tip stress concentration in LTIRs and enhance structural buckling resistance. Meanwhile, the gas trapped within closed pores provides positive pressure support under compressive loading, thereby suppressing the instability of solid skeletons and consequently improving the macroscopic mechanical properties of materials. As shown in Figure 3, Luan et al. fabricated closed-pore porous cordierite thermal insulation ceramics via the Pickering emulsion method with sucrose employed as the pore-forming agent. Owing to this closed-pore structure, the obtained samples achieved a compressive strength of 6.09 MPa and a minimum thermal conductivity of 0.4794 W/(m·K) at a porosity of 90.83% [37]. Huo et al. [38] fabricated closed-pore Al_2_O_3_/ZrO_2_-based thermal insulating ceramics exhibiting a superior cold compressive strength (CCS) of 1.11 ± 0.07 MPa at 96.7% porosity, outperforming previously reported open-cell Al_2_O_3_ LTIRs. Li et al. [39] adopted the same strategy to prepare Al_2_O_3_-based LTIRs with an even higher porosity of 97.49%, attaining a CCS of 0.76 MPa. Wang et al. [40] constructed closed-pore Al_2_O_3_-based thermal insulating ceramics via direct foaming and achieved a high CCS of 30 MPa at a porosity of 82%. Chen et al. [41] fabricated corundum-based hollow sphere LTIRs through foam gelcasting. Benefiting from the inherent closed-pore features of hollow spheres, a CCS of 1.81 MPa was achieved at a porosity of 80.2%.

Based on three-dimensional (3D) micro-CT reconstruction and finite element simulations, De Cet et al. [42] revealed that open and interconnected pores tended to trigger asymmetric bending and buckling instability under external loading. In contrast, closed pores transformed external loads into uniformly distributed circumferential membrane stress along pore walls, alleviating local tensile stress concentration and substantially elevating the structural load-bearing capacity. From the perspective of fracture mechanics, Li et al. [43] systematically compared the mechanical degradation behaviors of open-pore and closed-pore architectures and confirmed that closed pores induced far weaker deterioration in matrix strength compared with open pores. Furthermore, closed-pore structures remarkably improved the high-temperature mechanical performance of LTIRs. Deng et al. [44] synthesized closed-pore mullite thermal insulating ceramics that achieved a residual strength retention rate of 61% at a porosity of 67.7% after three air-cooling thermal cycles at 1000 °C. Xiang et al. [45] fabricated closed-pore (Zn_0.1_Ca_0.1_Sr_0.4_Ba_0.4_)ZrO_3_ thermal insulating ceramics, which exhibited a compressive strength of 0.97 MPa and low thermal conductivity of 0.086 W/(m·K) at a porosity of 92.3%. Their residual strength retention rate reached 77.8% after 10 thermal cycles, which was mainly attributed to crack deflection and crack pinning toughening. The above investigations demonstrated that closed-pore structures could simultaneously enhance the room-temperature and high-temperature mechanical properties of LTIRs.

In addition to pore connectivity, pore sphericity represents another crucial route for improving the mechanical performance of LTIRs. Owing to their minimum specific surface area and uniform curvature distribution, spherical pores alleviate stress singularity originating from irregular pore morphologies, facilitate uniform stress transfer around pore perimeters, and thereby delay crack initiation and propagation. The efficacy of this strengthening mechanism was verified in diverse refractory systems. Qian et al. [46] fabricated mullite LTIRs through gelcasting, using mullite powder and fly ash hollow spheres. The as-obtained refractories delivered higher CCS (6.21 MPa) at high porosity (72.2%). Liu et al. [47] constructed a high-sphericity pore structure in alumina-based LTIRs using spherical nano-sized carbon black as the pore-forming agent. The synthesized specimens exhibited a high porosity of 63%, CCS of 21.4 MPa, and a low thermal conductivity of 0.192 W/(m·K) at 300 °C. Remarkably, when compared to traditional counterparts with irregular pores, these refractories demonstrated superior high-temperature flexural strength and significantly higher residual strength retention following three air-cooling–thermal shock cycles. Subsequent optimization of pore sphericity was achieved via aluminum sol impregnation, which raised the compressive strength to 35.3 MPa while simultaneously enhancing the high-temperature flexural performance and TSR [48].

#### 4.1.2. Micro- and Nanoscale Pore Refinement: Mechanisms and Intrinsic Limits

According to Griffith’s brittle fracture theory (Equation (3)), the fracture strength of LTIRs is inversely correlated with the characteristic defect size, which in porous ceramics is physically represented by pore dimensions. Refining pore architectures down to the micro- or nanoscale therefore serves a dual mechanical function: it directly elevates the intrinsic fracture stress by minimizing fatal flaw dimensions, while simultaneously multiplying solid strut boundaries that induce extensive deflection, bifurcation, and pinning of advancing microcracks [49]. The efficacy of this refinement in enhancing ambient load-bearing capacity has been broadly validated. Under comparable porosities, Yang et al. [50] engineered microporous alumina ceramics with an MPS of 879.4 nm, achieving an impressive cold compressive strength (CCS) of 14.17 MPa at 82.91% porosity, while López et al. [51] utilized micro-scale pores (0.1~2 μm) to secure a CCS of 54 MPa in porous mullite with porosity of 56%. This microstructural strengthening pathway extends reliably to advanced ultra-high-temperature systems and operates under severe thermo-mechanical loading. Wang et al. [52] demonstrated that reducing the pore size from 1.63 to 0.78 μm in high-entropy rare-earth zirconates enhanced the compressive strength from 1.0 to 6.4 MPa. Despite their non-traditional nature, these high-entropy matrices display skeletal buckling behavior comparable to that of oxide LTIRs, highlighting pore refinement as a universal strategy to mitigate defect-driven failure (Figure 4). Zhao et al. [53] demonstrated that decreasing the mean pore size (MPS) from 181 to 54 μm yielded a nearly 200% increase in compressive strength via particle-stabilized foaming, even in ultra-high-porosity regimes (>80%).

Under high-temperature conditions, Liu et al. [54] verified that reducing the alumina MPS from 7.98 to 6.01 μm doubled the flexural strength at elevated temperatures from 3.1 to 6.2 MPa. Additionally, three-dimensional representative volume element (RVE) simulations by Shuai et al. [55] revealed that while coarse pores generate steep principal stress concentrations that act as premature failure origins, submicron and nanoscale pores induce only marginal perturbations in the thermal–mechanical stress field, thereby delaying macrocrack penetration and substantially improving TSR.

Despite these demonstrable strength advantages, an exclusive reliance on uniformly refined monomodal nanoscale pores incurs severe physical penalties during practical service. Homogeneous submicron frameworks inevitably yield high matrix stiffness (*E*), which escalates transient thermal stresses (*σ_f_*) and compromises the thermal shock damage resistance parameter (*R⁗*∝$E/\sigma_{f}^{2}$). Furthermore, uniformly fine skeletons lack compliance and crack-arresting void spaces, facilitating uninterrupted crack propagation once initiated. Thermally, ultrafine nanoporous skeletons generate substantial capillary driving forces for mass transport, accelerating grain coarsening, sintering shrinkage, and high-temperature creep during prolonged industrial campaigns. Consequently, monomodal pore refinement alone cannot resolve the intrinsic conflict among high mechanical strength, thermal barrier efficiency, and long-term damage tolerance.

#### 4.1.3. Hierarchical Multimodal Pore Architectures: Decoupling Thermal Insulation, Mechanical Strength, and Damage Tolerance

To resolve the zero-sum trade-off inherent to monomodal pore distributions, engineering cross-scale bimodal and multimodal hierarchical pore architectures has emerged as a state-of-the-art structural solution. In these optimized architectures, macroscopic pores (10~60 μm) are strategically coupled with submicron or nanoscale pores (50 nm~2 μm), which are integrated directly within the intercellular cell struts and boundaries [56].

As schematically illustrated in Figure 5, hierarchical bimodal networks synergistically decouple contradictory functional requirements across multiple physical regimes. Mechanically, as an advancing crack approaches an isolated macropore, the localized cluster of surrounding submicron pores induces extensive crack twisting, out-of-plane bifurcation, and crack-tip blunting [57]. These results indicate that the introduced micropores trigger crack deflection and effectively relieve pore-edge stress concentrations induced by isolated large pores. This tortuous dissipation path amplifies the effective work of fracture ($\gamma_{\mathrm{wof}}$) and substantially elevates the thermal shock damage resistance parameter ($R^{⁗}$) [58]:

(5) $$R^{⁗}=\frac{E_{\gamma_{wof}}}{\sigma_{f}^{2}\left(1-v\right)}$$

where *E* is Young’s modulus, $\sigma_{f}$ is the fracture strength, and *v* is Poisson’s ratio. Thermally, multimodal networks truncate heat transfer across wide temperature intervals: internal submicron pores suppress gas-phase conduction by activating the Knudsen effect (Lmean ≈ 70 nm for air at 25 °C), while dense internal boundaries multiply photon reflection and scattering to neutralize radiative transport (*K_rad_* $\propto d_{\mathrm{pore}}$*T*^3^, *K_rad_* is radiative thermal conductivity, $d_{\mathrm{pore}}$ is pore size, and *T* is temperature) above 1000 °C.

The efficacy of this hierarchical decoupling has been broadly verified by empirical investigations. Meng et al. [59] fabricated hierarchically porous La_2_Zr_2_O_7_ ceramics combining macropores (40~60 μm) and micro-cavities (0~10 μm) via particle-stabilized foaming, achieving a higher CCS of 3.6 MPa at 88% porosity. Similarly, Li et al. [60] synthesized hierarchical mullite ceramics via foam gelcasting, attaining a high CCS of 26.62 MPa coupled with a low thermal conductivity of 0.279 W/(m·K) at 75.7% porosity, which was directly attributed to pore-edge stress dilution and microcrack deflection. Exploiting an in situ Kirkendall-effect-driven oxidation route, Xia et al. [61] prepared micro-nano hierarchical hollow Al_2_O_3_ foams that retained a respectable CCS of 2.19 MPa even under an extreme porosity of 89.45%.

To rationally design such hierarchical cellular ceramics, a rigorous classification of pore connectivity states is essential, as distinct topologies generate divergent stress fields and degradation pathways under coupled service conditions (Table 3). Isolated closed pores convert uniaxial loads into circumferential membrane hoop stresses along cell walls—alleviating stress singularities, preventing strut buckling, and blocking Stage II corrosive slag and vapor ingress—though monomodal closed cavities offer limited nonlinear crack-arresting capability. Conversely, interconnected open pores promote asymmetric strut bending and buckling governed by the Gibson–Ashby power law (Equation (2)), while acting as express conduits for molten slag penetration and internal convective heat transfer. By strategically integrating closed, compliant macropores with tortuous micro–nanoporous struts, hierarchical multimodal networks effectively eliminate these separate drawbacks, simultaneously delivering high compressive load support, broad-temperature thermal insulation, and superior TSR.

### 4.2. Pore-Wall Densification and In Situ-Formed Bonding Phases

Beyond the regulation of pore structures, pore walls serve as the primary load-bearing structural units. Achieving pore-wall densification and bonding-phase strengthening constitutes an effective strategy to further enhance the mechanical performance of LTIRs. This objective can be realized via two main approaches. First, flux additives are incorporated to facilitate liquid-phase sintering. Defects inside pore walls are filled, and particle migration and rearrangement are accelerated, thereby achieving pore-wall densification. Second, in situ reactions are utilized to form thermally stable crystalline phases including mullite and spinel. The intrinsic matrix strength is improved by virtue of grain interlocking and grain-boundary pinning effects. The synergistic action of these two mechanisms allows the materials to achieve desirable mechanical properties even at high porosities.

#### 4.2.1. Pore-Wall Densification

Flux-triggered liquid-phase sintering represents a principal pathway for achieving pore-wall densification. Upon high-temperature treatment, the flux induces a eutectic liquid phase that infiltrates initial interparticle spaces and repairs microdefects inside the cell walls. This liquid-assisted process subsequently promotes neck growth and drives the final consolidation of the pore walls. For example, Huang et al. [62] fabricated porous thermal insulating ceramic aggregates via disk granulation using waste glass powder as the flux. Although the fabricated specimens reached a compressive strength of 5.3 MPa at 77% porosity, a further temperature rise to 1250 °C resulted in a dramatic strength loss down to 1.2 MPa, dictated by the drastic reduction in liquid viscosity that thinned the pore walls. Geng et al. [63] exploited the sintering-promoting effect of B_2_O_3_ to form mullite bonding phases on the surfaces of alumina hollow spheres. The resultant material delivered a CCS of 11.97 MPa at a porosity of 62.5%. Li et al. [64] employed Al_2_O_3_, SiO_2_ and MgO as raw materials to synthesize porous cordierite thermal insulating ceramics featuring 82.49% porosity and a high CCS of 2.6 MPa via the foam gelcasting method.

#### 4.2.2. In Situ-Formed Bonding Phases

Apart from flux-induced pore-wall densification, the mechanical performance of pore walls is further strengthened by the formation of interfacial bonding phases. Thermodynamically stable phases, including mullite, magnesium aluminate spinel, and TiB_2_, can be formed in situ on the pore walls of LTIRs. These phases effectively improve structural strength and service stability through multiple strengthening mechanisms such as grain interlocking, interface bridging, and Zener pinning. Yan et al. [65] fabricated mullite-based LTIRs with in situ-generated MgAl_2_O_4_ reinforcing phases on pore walls via a combination of reaction sintering and pore-forming technology. At a porosity of 41.11%, the synthesized material exhibited a high CCS of 107.17 MPa while maintaining 66.74% of its original strength following alkali attack. Bai et al. [66] prepared mullite-based thermal insulating ceramics through microwave sintering coupled with in situ reaction. The formed mullite phase bridged discrete SiC particles on the pore walls and built a continuous, integral skeleton. As a result, a superior combination of high flexural strength (125 ± 6 MPa) and suppressed thermal conductivity (6.52 W/(m·K)) was realized in the optimized material. Liu et al. [67] fabricated a mullite-based thermal insulating fiber composite with SiO_2_ as the bonding phase. The SiO_2_ particles strengthened intergranular connections, and the resultant material delivered a CCS of 1.22 MPa and thermal conductivity of 0.035 W/(m·K) at a porosity of 91.3%. The above investigations confirm that constructing thermodynamically stable bonding phases on pore walls via in situ reactions remarkably improves mechanical performance and corrosion resistance while maintaining high porosity. This strategy constitutes a promising pathway for the compositional optimization of LTIRs.

### 4.3. Synergistic Toughening and Strengthening via Cross-Scale Reinforcements

Apart from reinforcement phases formed on pore walls, introducing secondary phases or reinforcements into the solid matrix represents another effective strategy to improve the mechanical performance of LTIRs. The commonly adopted reinforcing components mainly include particles, whiskers, and fibers.

#### 4.3.1. Particle Reinforcement

For particle-type second-phase strengthening and toughening systems, harnessing the phase transformation of ZrO_2_ to enhance both strength and fracture toughness constitutes a widely adopted strategy. This toughening is mainly achieved through a 3~5% lattice expansion associated with the stress-induced martensitic transformation of metastable tetragonal ZrO_2_ into the monoclinic structure at crack propagation fronts. The compressive stress layer formed during phase transformation effectively shields tensile stress at crack tips and dissipates fracture energy, thereby restraining continuous crack propagation (Figure 6).

Shi et al. [69] utilized zirconium sol as the precursor of reinforcing phases to fabricate Ca_2_Mg_2_Al_28_O_46_ (CMA)-based porous ceramics. Driven by ZrO_2_ phase transformation and crack deflection mechanisms, the developed material sustained 66.4% of its original strength even at an ultra-high porosity of 85.06%. Liu et al. [70] prepared ZrO_2_-toughened porous Al_2_O_3_-based thermal insulating ceramics using a dual-phase nanoparticle-stabilized foaming route. Thermal annealing at 1200 °C transformed the zirconium sol into uniformly distributed ZrO_2_ nanoparticles, which imparted superior mechanical strength, effective thermal insulation, and enhanced TSR to the material. Even with an elevated porosity reaching 86.7%, the synthesized material demonstrated an excellent balance between mechanical support and thermal insulation, delivering a CCS of 4.7 MPa along with a low thermal conductivity of 0.092 W/(m·K). Increasing the ZrO_2_ loading further improved the residual strength retention rate upon repeated thermal shocks. Apart from single particle reinforcement, numerous studies have explored dual-phase synergistic toughening mechanisms. Zake-Tiluga et al. [71] incorporated nano-sized SiO_2_ and SiC particles as secondary phases into lightweight insulating alumina materials to impede crack propagation, thereby enhancing the mechanical properties and decreasing thermal conductivity of lightweight ceramics. This finding provides a novel strategy for the development of high-strength thermal insulating ceramics.

#### 4.3.2. Strengthening and Toughening via Whiskers/Fibers

In addition to the transformation toughening effect of particulate second phases, the incorporation of whiskers or fibers serves as another vital strategy to enhance the mechanical performance of LTIRs [72]. These high-aspect-ratio reinforcements construct continuous load-bearing skeletons within the matrix. Interfacial shear stress facilitates effective load transfer and alleviates the tensile stress sustained by the matrix. As shown in Figure 7, energy dissipation during fracture is primarily governed by a combination of crack deflection, interfacial debonding, fiber pull-out, and crack bridging. These concurrent mechanisms effectively hinder crack propagation, culminating in a simultaneous enhancement of both mechanical strength and fracture toughness.

Bai et al. [73] fabricated ZrO_2_ whisker-reinforced porous thermal insulating material via freeze-drying. They confirmed that the incorporation of ZrO_2_ whiskers effectively suppressed the tetragonal-to-monoclinic phase transformation of ZrO_2_ and significantly improved flexural strength. Qin et al. [74] prepared mullite fiber-reinforced cordierite LTIRs with porosity of 58.9% by the gelcasting method. The synthesized material maintained a respectable CCS of 10.0 MPa while offering effective thermal conductivity of 0.342W/(m·K) (1000 °C). Liu et al. [75] fabricated whisker-reinforced mullite-based LTIRs by using carbonized rice husk as a pore-forming agent. Benefiting from the 3D interlocked whisker architecture, the synthesized material exhibited superior CCS (45.0 MPa), relatively low thermal conductivity (0.189 W/(m·K)), and high porosity (74.3%). Furthermore, the specimen displayed desirable TSR. Liu et al. [76] prepared alumina-based cellular ceramics bonded by an in situ-formed mullite phase using Al_2_O_3_-Si-boehmite particle-stabilized foams. Benefiting from crack inhibition originating from whisker/fiber rupture and pull-out, the resulting sample possessed the robust compressive strength of 2.26 MPa at a high porosity of 87.8%.

### 4.4. Heterogeneous Interface Engineering

The aforementioned studies demonstrate that introducing reinforcing phases can substantially improve the mechanical performance of LTIRs while preserving high porosity via synergistic interactions among various toughening mechanisms. Nevertheless, the addition of reinforcements inevitably modifies both pore architecture and interfacial bonding. Under extreme service environments featuring rapid heating and cooling, enormous temperature gradients, and erosion from complex corrosive media, three types of interfaces inside LTIRs—matrix/bonding phase interfaces, reinforcement/pore-wall interfaces, and multiphase sintered interfaces—are prone to severe localized stress concentration originating from thermal mismatch. These interfaces thus act as vulnerable sites that trigger premature structural spalling and performance degradation [77,78]. The interfacial thermal stress *σ_th_* is given as follows (Equation (6)) [79]:

(6) $$\sigma_{\mathrm{th}}=f(P)\frac{E_{A}E_{B}}{E_{A}(1-\upsilon_{B})+E_{B}(1-\upsilon_{A})}\left|\alpha_{A}-\right.\left.\alpha_{B}\right|\Delta T$$

where $\alpha_{A}$ and $\alpha_{B}$ are the TEC of the heterogeneous phases, respectively. The *f*(*P*) is a stress attenuation factor that depends on the porosity *P* and pore structure (typically *f*(*P*) < 1). The *σ_th_* is the interfacial thermal mismatch stress; *E_A_* and *E_B_* are the elastic modulus of the *A* and *B* phases, respectively. The *υ_A_* and *υ_B_* are the Poisson’s ratios of the *A* and *B* phases, respectively. △*T* is the amplitude of temperature change. Constructing porous interfaces capable of buffering thermal stress via multi-scale synergistic regulation of pores, pore walls and heterogeneous interfaces constitutes the key to the simultaneous improvement of high porosity, strength-toughness and TSR. Accordingly, researchers globally have performed systematic interfacial engineering designs based on three core mechanisms: the fabrication of continuously graded interfacial transition layers, energy dissipation through weakly bonded interfacial sliding, and viscoplastic flow of intergranular glassy phases at ultra-high temperatures.

#### 4.4.1. Continuously Graded Interfacial Transition Layer

Constructing compositionally graded interfacial transition zones on the pore-wall surfaces of LTIRs via in situ interdiffusion and solid-state reactions effectively bridges abrupt discontinuities in the coefficient of thermal expansion and elastic modulus between adjacent phases [80]. By smoothing out interfacial property gradients, this architecture lowers the localized peak thermal mismatch stress and suppresses the nucleation and propagation of interfacial cracks under thermal cycling.

The efficacy of this strategy has been demonstrated in several high-performance multiphase refractory systems. For instance, Wang et al. [81] incorporated alumina powder to engineer a graded anorthite–alumina–spinel transition layer in porous anorthite–spinel refractories. Benefiting from the viscoelastic stress relaxation of this tailored transition zone at elevated temperatures, interfacial thermal stresses were efficiently buffered, yielding an enhanced CCS of 1.84 MPa and a low thermal conductivity of 0.213 W/(m·K) at 1000 °C at 76.1% porosity. Similarly, Jin et al. [82] engineered an in situ ZrO_2_ transition layer at the corundum–spinel interface in Al_2_O_3_-MgAl_2_O_4_ lightweight castables. This buffer layer effectively accommodated modulus discrepancies, arrested microcrack deflection, and preserved interfacial bonding integrity across a broad thermal window, thereby markedly elevating the TSR of the refractory framework.

Extending beyond high-purity chemical routes, solid-waste upcycling (e.g., coal gangue, red mud, and fly ash) provides an economically attractive approach for in situ gradient interface engineering. The complex multi-oxide constituents (SiO_2_, Al_2_O_3_, CaO and MgO) inherent to these industrial byproducts undergo spontaneous multi-element diffusion and non-equilibrium reactions during high-temperature sintering, naturally forming multilevel graded interfaces along pore walls [83,84]. These endogenously formed transition layers not only mitigate thermal stress concentrations without requiring expensive synthetic additives but also significantly reduce raw-material costs. Nevertheless, addressing batch-to-batch compositional variations and establishing industrial-scale processing stability remain critical challenges for the widespread commercialization of these waste-derived, high-strength LTIRs.

#### 4.4.2. Weakly Bonded Interface Design

Unlike conventional strategies pursuing interfacial bonding, the exploitation of moderate weak bonding at heterogeneous interfaces to induce size-controllable microcrack arrays and interlayer sliding under thermal shock loading represents a critical nonlinear toughening route to enhance the TSR of porous thermal insulation ceramics. In this regard, Muto et al. [85] fabricated alumina LTIRs with a house-of-cards structure by introducing alumina platelets together with a gelatin-sol (as binder) and freeze-drying technique. As thermal stress waves propagated toward these weak interfaces, the material exhibited porosity of 80.2%, CCS of 1.7 MPa, and thermal conductivity of 0.94 W/(m·K). Sakihama et al. [86] introduced plate-like calcium hexaluminate (CA_6_) formed by an in situ reaction in porous alumina thermal insulating ceramics to construct controllable weak interlayer sliding interfaces. The synthesized material achieved a superior CCS (1.1 MPa) and relatively low thermal conductivity (0.074 W/(m·K)), even at an ultra-light porosity (85%). Furthermore, the stable platelet microstructure effectively promoted the crack deflection and arrest, consequently achieving a prominent enhancement in thermal shock resistance. Similarly, Shimamura et al. [87] induced highly oriented plate-like grains at the surface of alumina LTIRs, forming weakly bonded interfaces with the porous ceramic surface. Owing to the unique two-dimensional lamellar van der Waals weak interfacial structure, a multistage synergistic energy dissipation and toughening mechanism was realized. Through microstructural interface-tailored regulation and stress dissipation, this approach successfully boosted the material’s fracture toughness along with its structural stability under prolonged thermal shock.

#### 4.4.3. Viscoelastic Flow of Grain-Boundary Glassy Phases

LTIRs encounter significant challenges in achieving high TSR under ultra-high-temperature service conditions exceeding 1200 °C. In recent years, precisely modulating the thickness and chemical composition of intergranular glassy films (IGFs), combined with harnessing the viscoelastic flow of glassy phases at ultra-high temperatures to relieve interfacial thermal stress, has emerged as a highly promising dynamic self-toughening strategy for high-temperature applications. Zheng et al. [88] investigated lightweight coal gangue-based (anorthite–mullite multiphase) thermal insulating bricks and revealed that coal gangue facilitated the formation of high-viscosity, silica-rich glassy phases at elevated temperatures (Figure 8). These glassy phases alleviated interfacial thermal stress and further enhanced the CCS of the materials. Chen et al. [89] investigated porous CaO-Al_2_O_3_-SiO_2_ thermal insulation ceramics and revealed that CaO facilitated the formation of high-viscosity, glassy phases at elevated temperatures. The as-synthesized material exhibited a favorable CCS of 6.8 MPa, a low thermal conductivity of 0.109 W/(m·K), and high porosity of 62%. These glassy phases mitigate interfacial thermal stress, thereby further enhancing the TSR of the material. However, Höfling et al. [90] noted that the IGF thickness should be regulated within an appropriate nanoscale range, with approximately 10 nm serving as a critical threshold. Excessively thick IGFs degraded high-temperature creep resistance and might even induce softening and collapse of the porous framework, whereas ultra-thin IGFs failed to provide efficient stress relaxation. Collectively, constructing controllable viscoelastic stress-relaxation interfaces in porous LTIRs through nanoscale structural design and compositional optimization of IGFs offers an effective route to boost ultra-high-temperature thermal shock and spallation resistance. The crux of this approach lies in achieving a delicate balance between IGF thickness and chemical composition, which ensures stable long-term service of the materials under severe thermal shock environments.

### 4.5. Synergistic Mechanisms of Multi-Strategy Integration

As summarized in Table 4, although individual strengthening routes enhance specific mechanical properties of LTIRs, each standalone approach remains constrained by intrinsic performance trade-offs. Consequently, realizing structural–functional integration—simultaneously reconciling high porosity, load-bearing capacity, and thermal shock damage tolerance—demands the multi-scale synergistic integration of complementary strengthening mechanisms across diverse service regimes:

(1) System-tailored strategy implementation: Strengthening protocols must align with the intrinsic thermophysical profiles and failure mechanisms of specific matrices. For high-expansion alumina- and zirconia-based systems, coupling nano-ZrO_2_ transformation toughening with weakly bonded sliding interfaces accommodates thermal-mismatch stresses and suppresses catastrophic thermal-shock cracking. Conversely, for low-expansion silicate- and cordierite-based ceramics, in situ whisker growth and nanoscale IGFs regulation are paramount to reinforce thin pore walls against buckling while averting high-temperature softening and alkali corrosion.

(2) Structural-reinforcement synergy: Closed-pore spherical micropores serve as the structural skeleton, within the pore walls of which whiskers or networked secondary phases are grown in situ. This configuration retains the efficient phonon-scattering effect of the closed-pore architecture, while the crack-bridging and fiber pull-out effects of the whiskers substantially improve the intrinsic fracture toughness of the pore walls. Such a design thus achieves the integrated optimization of a lightweight skeleton and localized structural strengthening and toughening.

(3) Double-edged sword regulation of solid waste systems: For industrial solid wastes such as coal gangue and red mud, impurity phases (e.g., Na_2_O and Fe_2_O_3_) are deliberately converted, through precise compositional control, into chemical driving forces that induce liquid-phase sintering and the in situ formation of binding phases (e.g., mullite and spinel). Coupled with gradient interface engineering, this strategy simultaneously enables the synergistic optimization of material properties and a reduction in raw material costs.

(4) Synergistic stress response across a wide temperature range: Achieving full-temperature damage tolerance relies on the sequential coordination of three interfacial mechanisms: graded transition layers mitigate thermal mismatch stress at moderate temperatures; weakly bonded interfaces activate sliding-induced energy dissipation during thermal shock; and the viscoelastic flow of IGFs enables dynamic stress relaxation and self-toughening above 1200 °C.

## 5. Conclusions and Perspectives

### 5.1. Conclusions

In this survey, the breakdown pathways of lightweight thermal insulation refractories (LTIRs) under intricate multi-physical field coupling are rigorously clarified, the intrinsic physics underlying the strength–insulation trade-off is uncovered, and mechanical reinforcement approaches are broadly categorized across four distinct dimensions: micro-pore architecture tailoring, pore-wall optimization, multi-scale reinforcement, and heterogeneous interface engineering. The primary conclusions are presented as follows:

(1) Multi-field coupled degradation mechanisms. Mechanical degradation in LTIRs is fundamentally driven by reduced solid load-bearing areas and severe pore-boundary stress concentrations under harsh service. Failure progresses through a three-stage sequence: microcrack initiation and coalescence, corrosion-induced volume distortion, and creep-driven skeletal collapse. Since degradation pathways are highly system-dependent, strengthening strategies must be tailored to individual systems: nano-ZrO_2_ transformation toughening mitigates thermal mismatch stresses in high-expansion alumina/zirconia systems, whereas in situ reaction engineering and modulation of viscoplastic IGFs play a critical role in low-expansion silicate/cordierite matrices.

(2) Micro-pore architecture tailoring. Closed-pore structures prevent the propagation of stress concentrations at crack tips, while spherical pores attenuate stress-inhomogeneous distributions induced by irregular pore geometries. Refining pore sizes to micro/nano scales reduces critical defect dimensions, whereas hierarchical porous architectures promote crack deflection and branching. These strategies significantly enhance mechanical strength and thermal shock stability while maintaining a higher porosity level.

(3) Pore cell-wall densification and multi-scale reinforcement toughening. Flux-induced liquid-phase sintering promotes pore-wall densification, while in situ-formed reinforcing phases (e.g., mullite, magnesium aluminate spinel) can enhance intrinsic pore-wall strength via grain interlocking and pinning effects. Furthermore, multiple energy-dissipation mechanisms, including phase transformation toughening of ZrO_2_ particles, as well as crack bridging, pull-out, and deflection caused by whiskers or fibers, synergistically improve both skeletal strength and fracture toughness.

(4) Heterogeneous interface engineering. At intermediate-to-low temperatures, continuous gradient interfacial transition zones mitigate thermal mismatch stress peaks. During thermal shock, weakly bonded interfaces facilitate microcrack sliding for energy dissipation. Above 1200 °C, the viscoplastic flow of intergranular glass phases associated with phase transformations can enable interfacial self-healing. Operating synergistically across a broad temperature domain, these mechanisms substantially increase thermal shock damage tolerance.

### 5.2. Perspectives

Despite significant progress in tailoring the mechanical and thermal performances of LTIRs, existing research has predominantly centered on isolated structural units or single-component modifications. A comprehensive quantitative design methodology and long-term service evaluation framework under multi-physical field coupling remains noticeably absent. Driven by the urgent demand for structural–functional integration in next-generation high-temperature equipment, future research should prioritize the following strategic directions:

(1) Multi-field coupled in situ dynamic characterization and standardized benchmark databases. Future efforts must transcend ambient, post mortem evaluations by deploying advanced in situ diagnostic facilities under coupled thermo-mechano-chemical environments. Integrating synchrotron radiation X-ray micro-CT, high-temperature digital image correlation (DIC), and acoustic emission enables real-time, non-destructive tracking of damage kinetics across the full life cycle—capturing transient thermal-stress cracking (stage I), chemical degradation and volumetric dilation (stage II), and long-term creep-induced skeletal collapse (stage III). Crucially, characterization protocols should advance beyond reporting scalar total porosity to quantitatively resolve topological metrics, including 3D pore connectivity, sphericity distributions, and multimodal cell-strut dimensions. Establishing standardized, open-access experimental datasets that correlate quantified residual stress profiles, chemical ingress depths, and high-temperature creep rates with processing histories is essential for calibrating predictive constitutive models.

(2) Physics-informed cross-scale modeling and machine-learning-driven inverse design. A unified multi-scale computational framework spanning atomic to macro scales is imperative to accelerate material exploration. High-throughput first-principles calculations and molecular dynamics can quantify interfacial bonding and grain-boundary mobility, feeding constitutive parameters directly into continuum damage mechanics (CDM), phase-field fracture models, and representative volume element (RVE) thermo-mechanical simulations. Concurrently, developing physics-informed neural networks (PINNs) and generative AI architectures (e.g., diffusion models) trained on high-fidelity multi-physical simulation datasets can effectively resolve the multi-objective Pareto trade-offs among porosity, thermal conductivity, and fracture energy. Such surrogate modeling frameworks will enable the rapid inverse design of hierarchical pore topographies and heterogeneous interfaces tailored for predefined industrial target envelopes.

(3) Multilevel composite interfaces and high-entropy multi-principal component engineering. Developing hierarchical interfacial architectures that integrate compositionally graded transition layers, weakly bonded two-dimensional (2D) interfaces (e.g., platelet CA_6_ or tabular corundum), and nanoscale intergranular glassy films remains a promising pathway to achieve dynamic stress relaxation across broad service-temperature windows. Simultaneously, introducing high-entropy designs (e.g., multicomponent rare-earth zirconates, silicates, and aluminates) into solid skeletons or interfacial phases provides unique compositional degrees of freedom. Exploiting the severe lattice distortion and sluggish cooperative diffusion inherent to high-entropy lattices can fundamentally delay high-temperature grain coarsening, suppress creep deformation, and disrupt phonon propagation pathways without relying on excessive cellular void volume.

(4) Industrial design roadmaps and life-cycle reliability evaluation. While strategy integration provides a conceptual foundation for property optimization (Figure 9), bridging laboratory synthesis to industrial deployment requires robust engineering roadmaps. Future work should establish multivariable design diagrams that prioritize strengthening routes based on dominant service regimes—balancing high-temperature compressive creep against transient thermal shock spallation. Furthermore, when compiling engineering databases, strict distinctions must be maintained between structural forms (e.g., bulk bricks, castables, monolithic coatings, and flexible aerogels). Finally, incorporating cost–benefit analyses, batch-to-batch stability metrics from solid-waste utilization, and life-cycle carbon footprint assessments will be indispensable to deliver sustainable, ultra-reliable LTIRs for green thermal infrastructure.

## Figures and Tables

**Figure 1:**
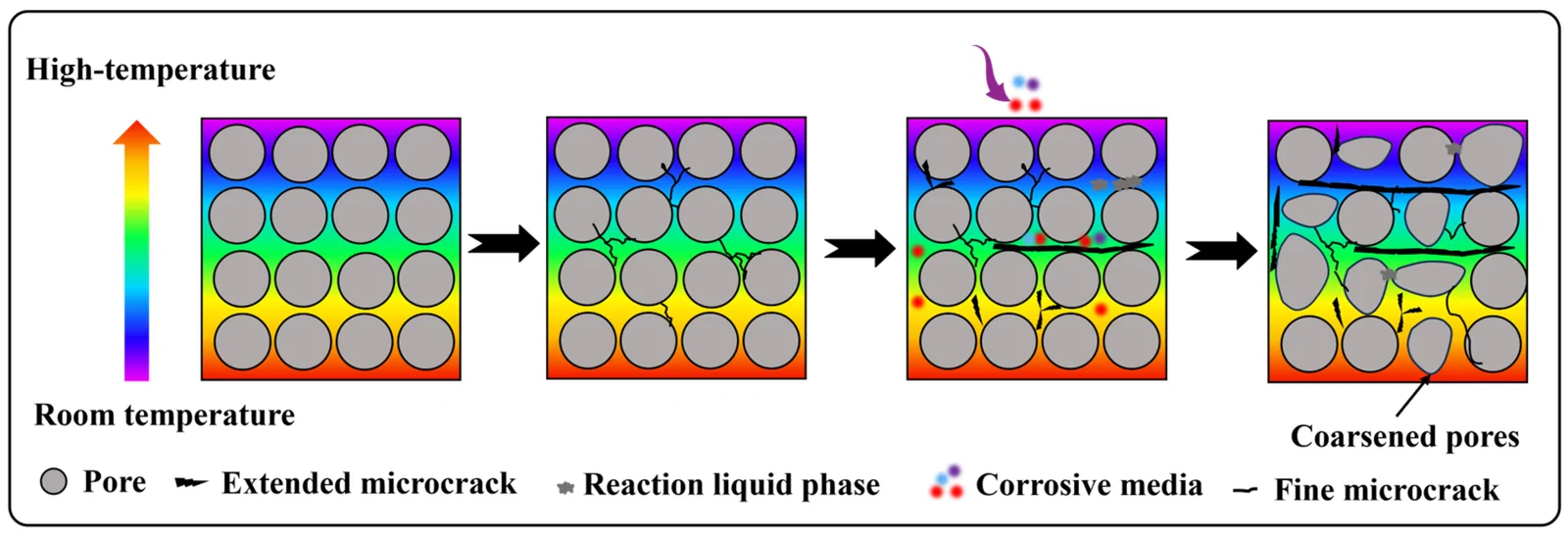
Schematic illustration of the three-stage progressive degradation mechanism for LTIRs under coupled thermo-mechano-chemical service conditions. Drawn by the authors.

**Figure 2:**
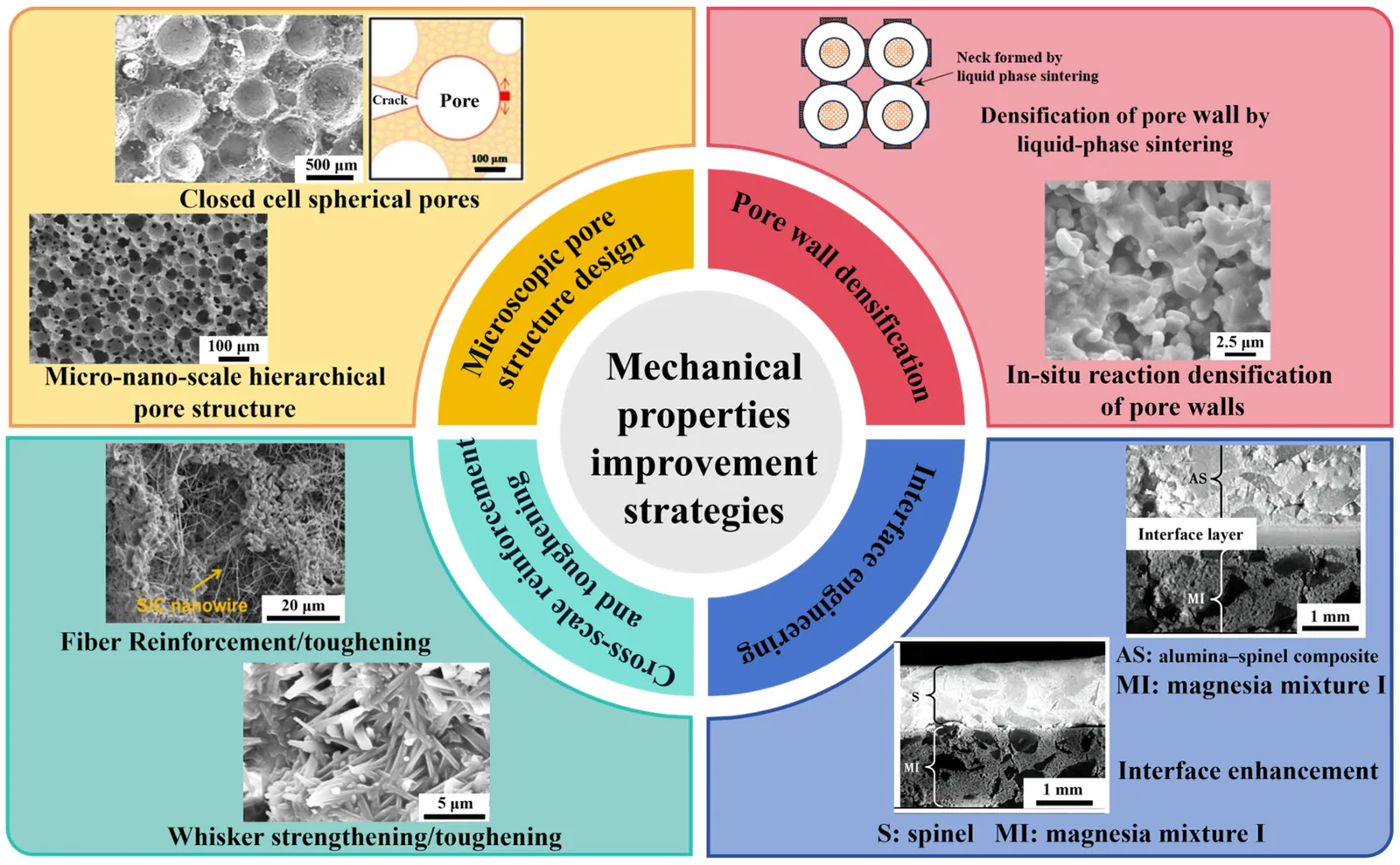
Schematic of four strategies for improving the mechanical properties of LTIRs: pore structure design, pore-wall densification, cross-scale reinforcement-toughening, and heterogeneous interface engineering. Adapted with permission from Refs. [32,33,34,35,36].

**Figure 3:**
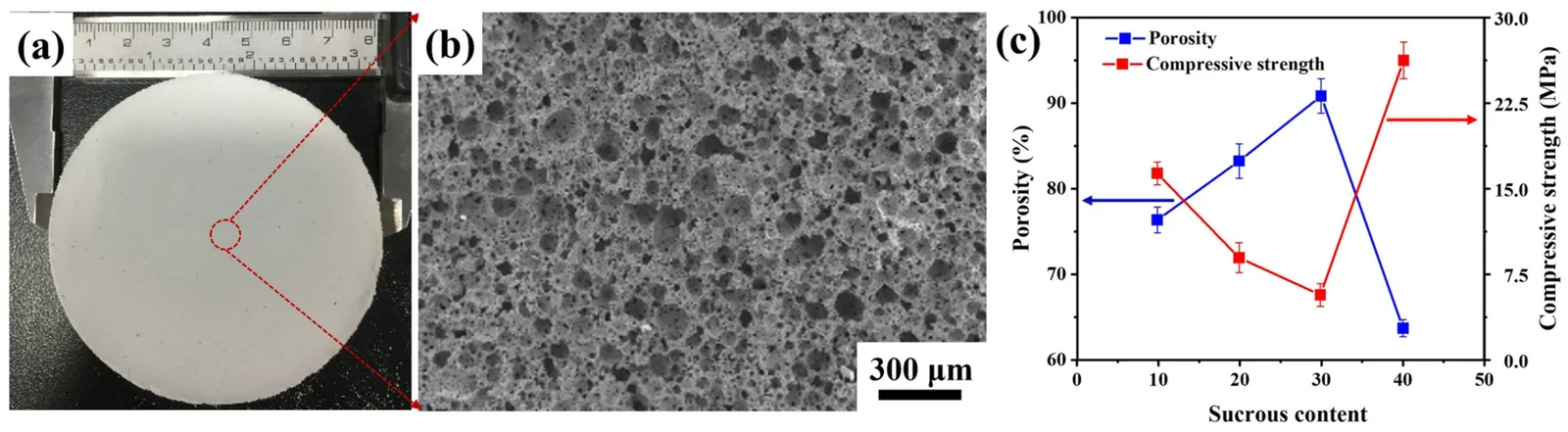
(**a**) Digital photographs, with a red dashed arrow indicating the region magnified in (**b**); (**b**) SEM images, and (**c**) porosity (blue line, left Y-axis) and compressive strength (red line, right Y-axis) of porous cordierite thermal insulating ceramics containing spherical pores. Reproduced with permission from Ref. [37].

**Figure 4:**
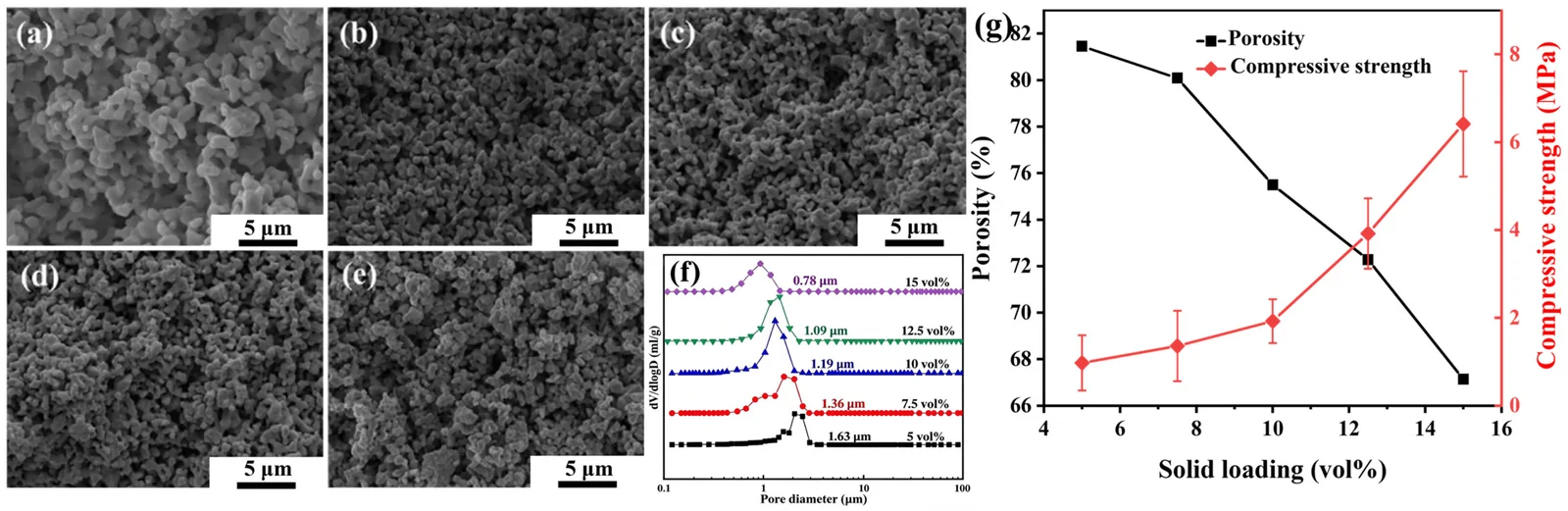
(**a**–**e**) Microstructures, (**f**) pore size distribution, (**g**) porosity, and compressive strength of high-entropy rare-earth zirconate thermal insulating ceramics containing micro-sized pores with different solid loadings. Reproduced with permission from Ref. [52].

**Figure 5:**
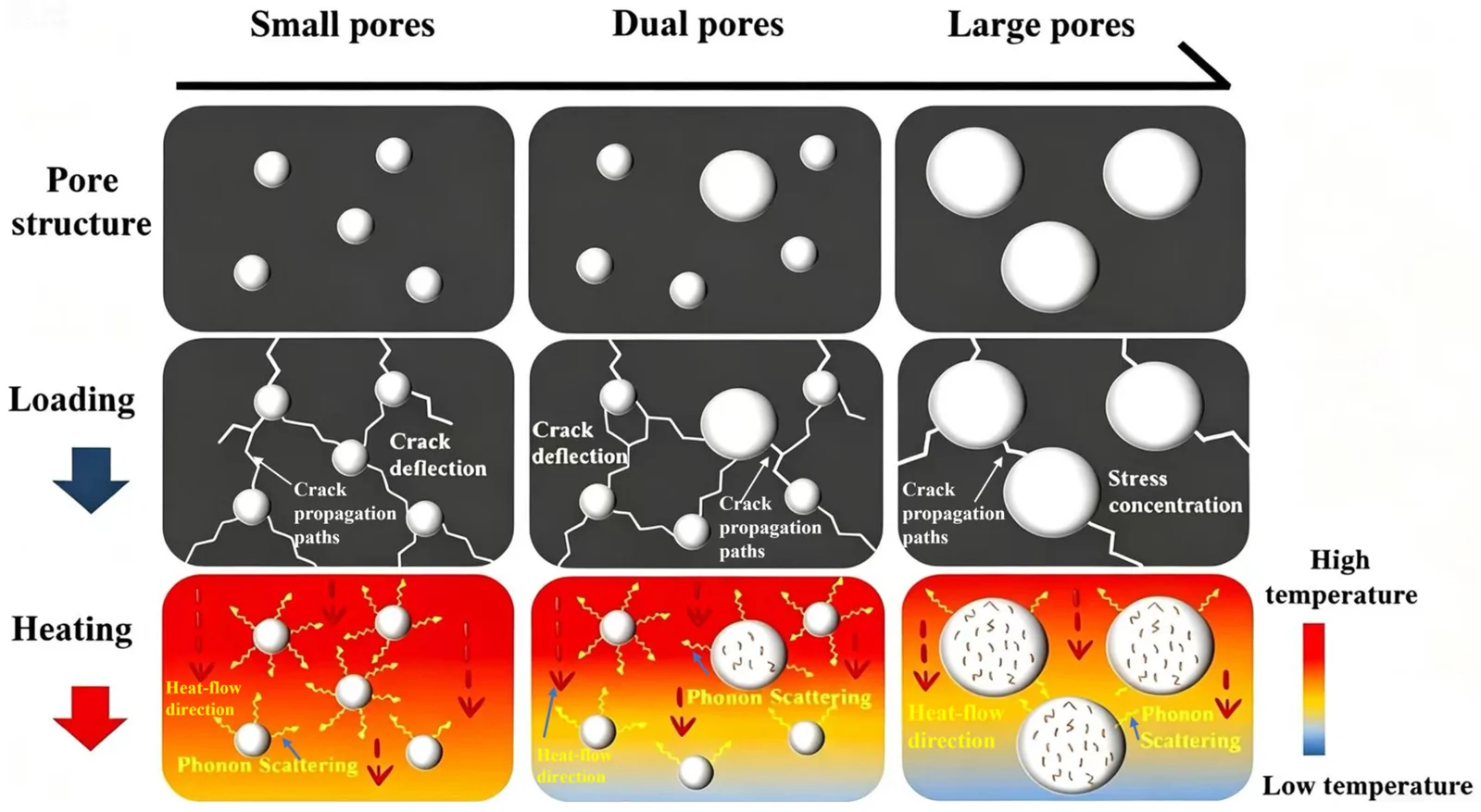
Schematic illustration comparing mechanical load transfer, crack propagation pathways, and thermal transport mechanisms among unimodal small pores, dual-scale hierarchical pores, and unimodal large pores in porous thermal insulating ceramics. Adapted with permission from Ref. [57].

**Figure 6:**
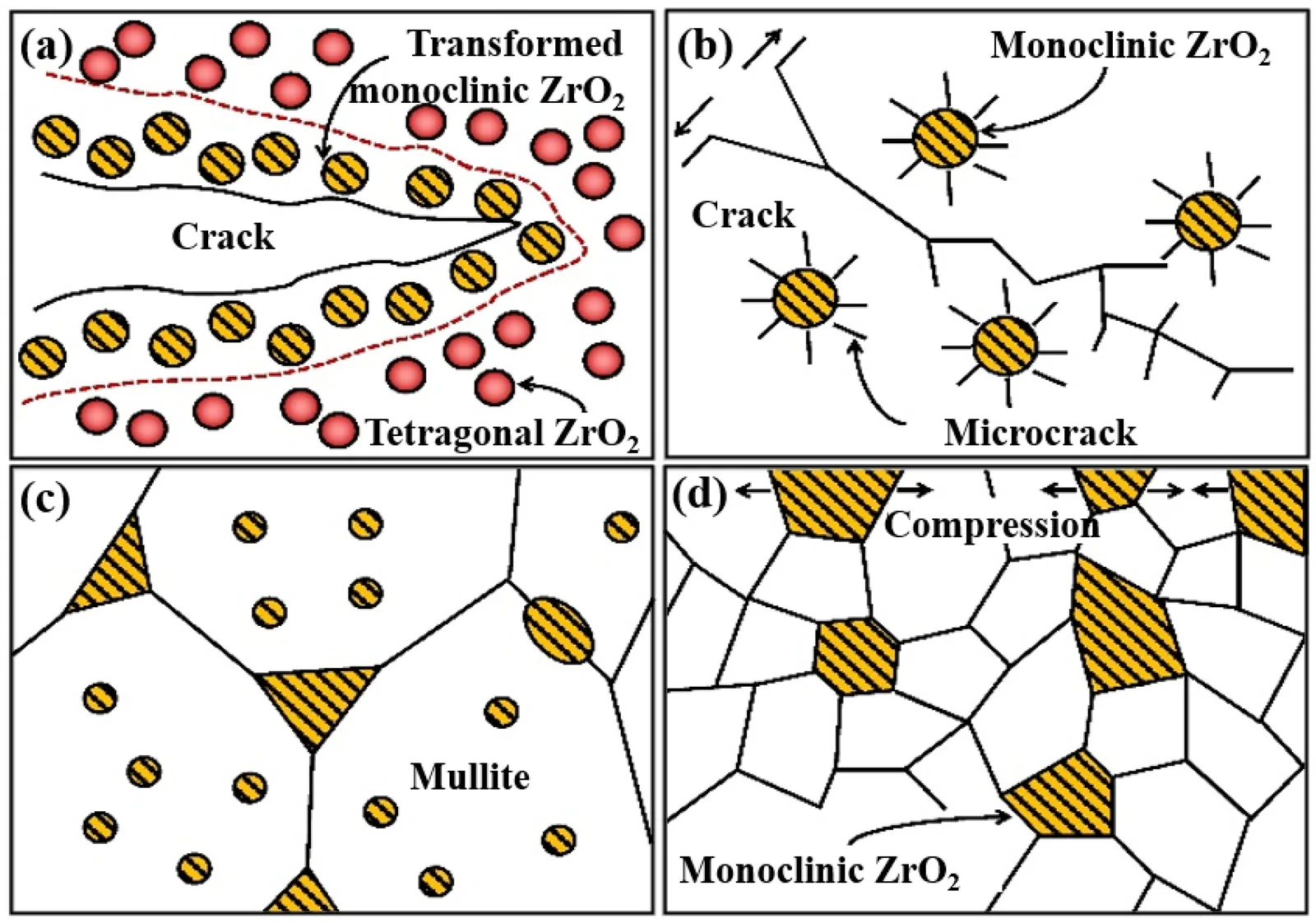
Schematic of energy dissipation and toughening mechanisms facilitated by ZrO_2_ particles in a mullite ceramic matrix: (**a**) stress-induced toughening: black solid lines represent crack paths, while red dashed lines outline the transformation zone (process zone), where tetragonal ZrO_2_ (red circles) transforms into monoclinic ZrO_2_ (hatched yellow circles). (**b**) microcrack toughening: black lines indicate the main crack and radiating microcracks. (**c**) ZrO_2_ incorporation toughening: black lines denote grain boundaries. (**d**) surface compression toughening: black solid lines represent compressive stresses. Adapted with permission from Ref. [68].

**Figure 7:**
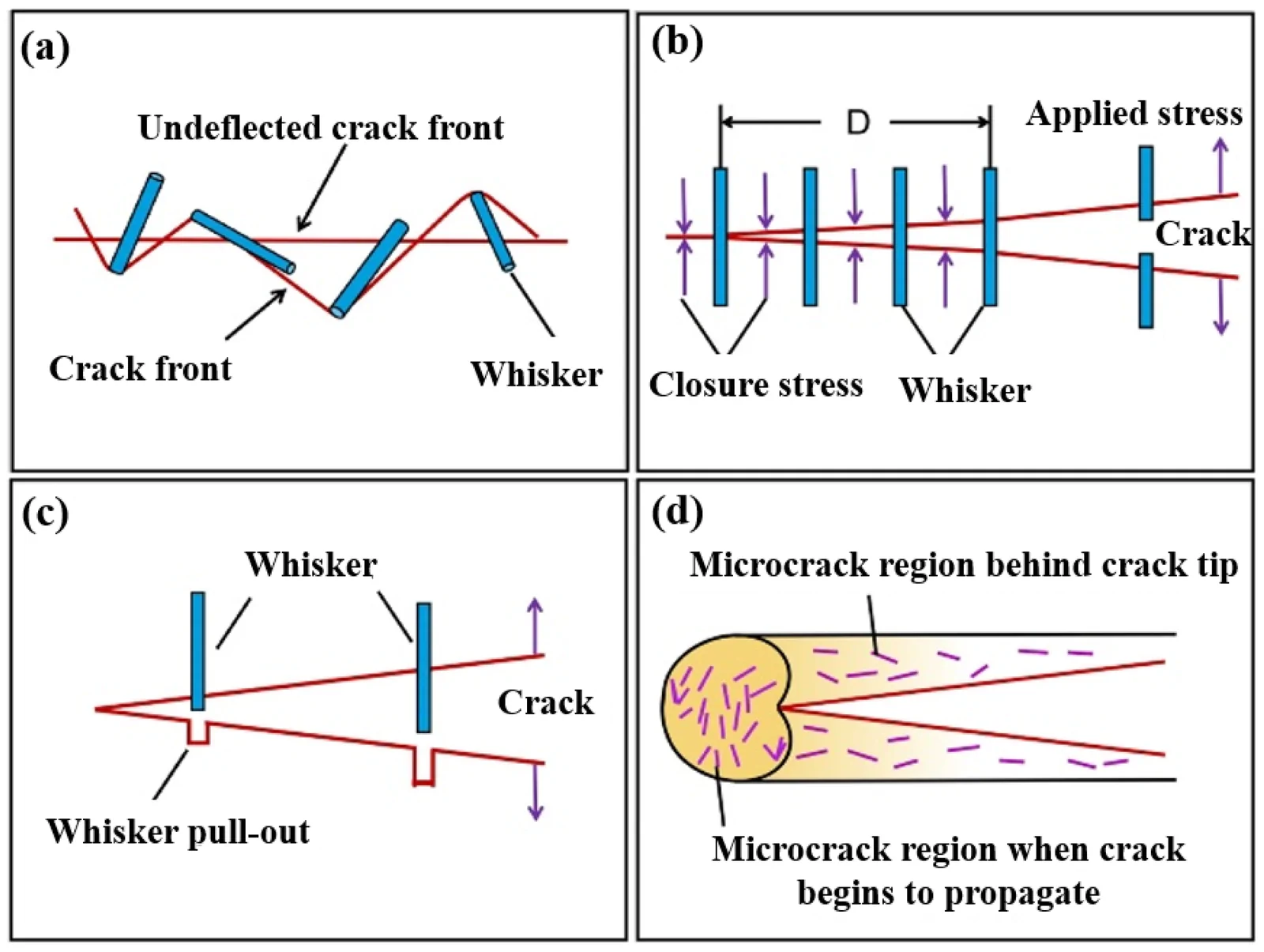
Schematic diagram of the toughening mechanisms of whiskers: (**a**) mechanism of crack deflection; (**b**) mechanism of crack bridging; (**c**) Scheme of whisker pull-out mechanism; and (**d**) mechanism of microcrack propagation. Adapted with permission from Ref. [68].

**Figure 8:**
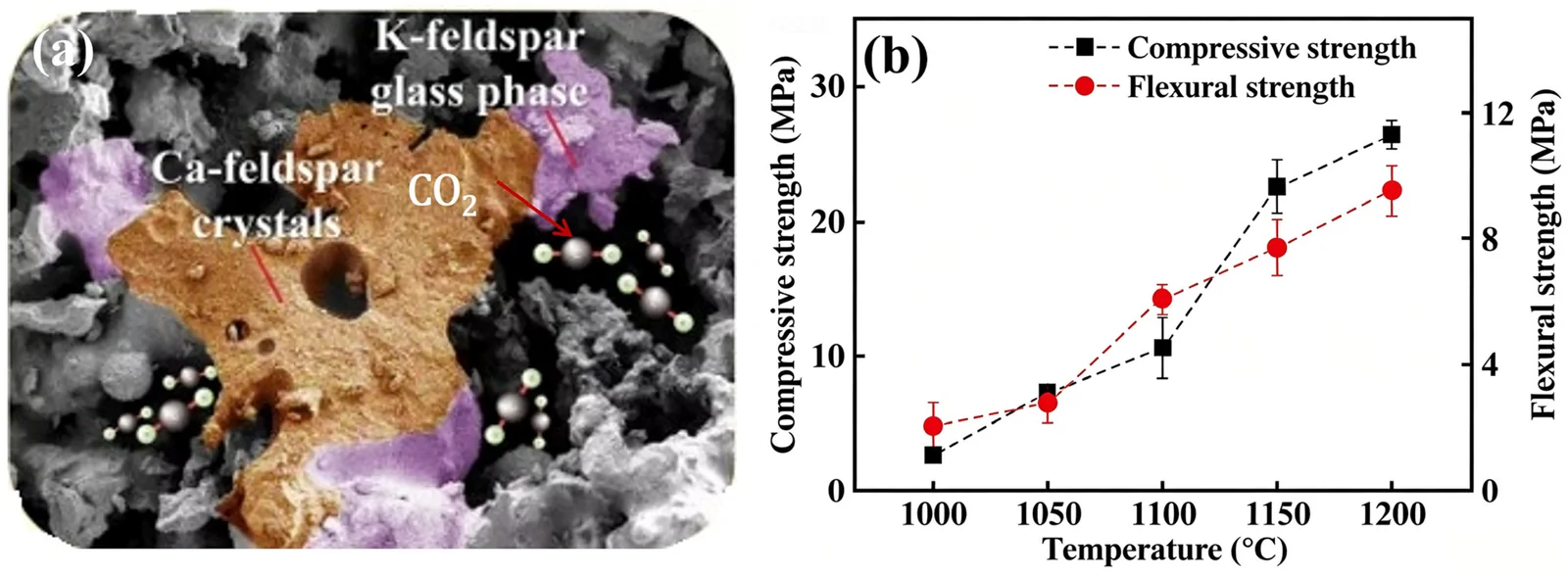
(**a**) Microstructures and (**b**) temperature-dependent mechanical performance of coal gangue-based anorthite–mullite multiphase insulation bricks containing a glass phase. Reproduced with permission from Ref. [88].

**Figure 9:**
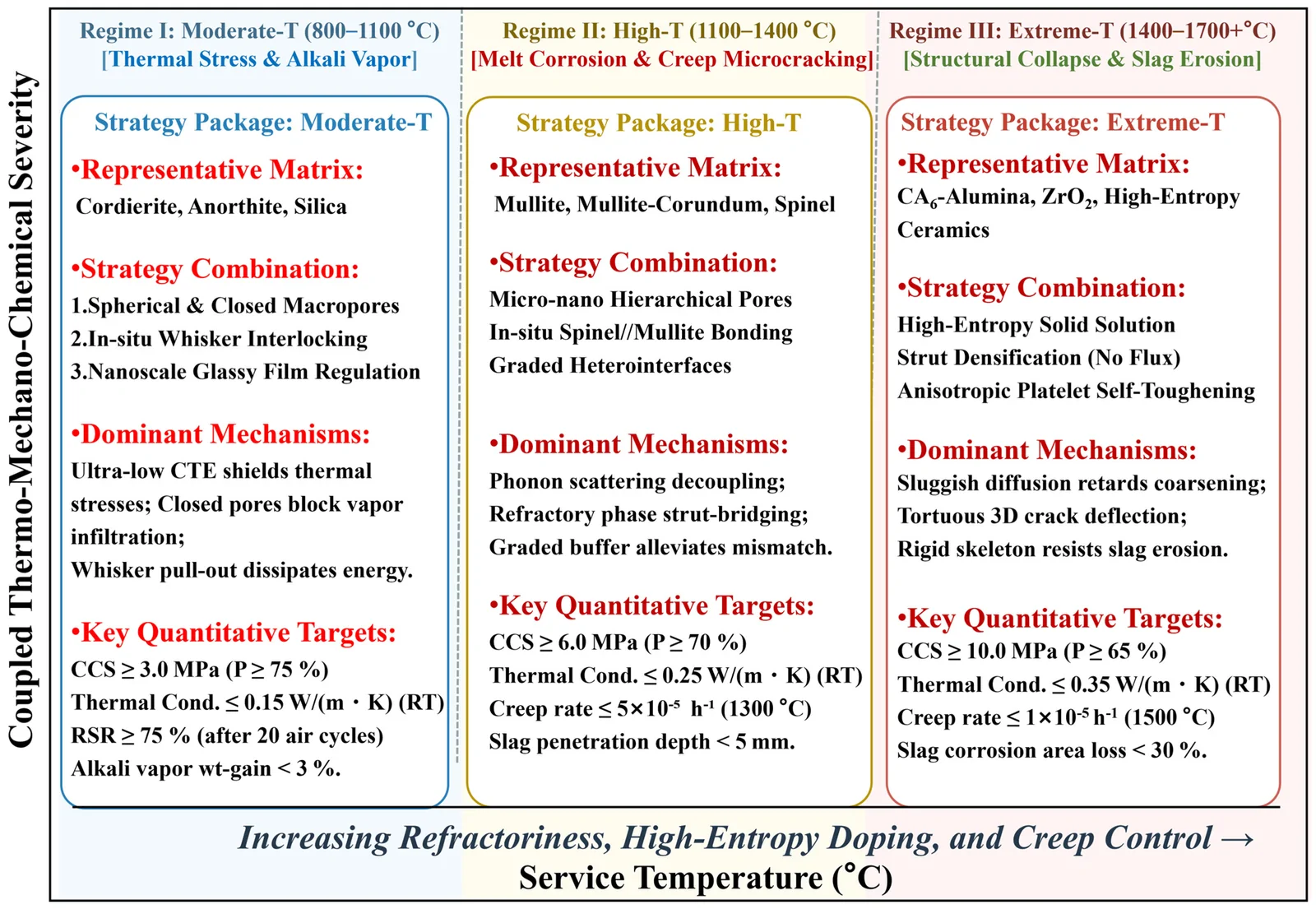
Service-condition-based design map and technical target roadmap for LTIRs. Regimes I-II correspond to bulk oxide-based LTIR matrices; Regime III also includes porous ultra-high-temperature ceramic candidates for extreme-condition applications. Drawn by the authors.

**Table 1:** Comparison of this review with previous related reviews.

Review Article	Focus Area	Key Contributions	Limitations/Gaps	References
Reviews on porous ceramics (general)	Processing-structure–property relationships of porous ceramics	Comprehensive coverage of fabrication methods and fundamental properties	No specific focus on refractory service environments; limited discussion of degradation mechanisms	[14]
Reviews on thermal insulation refractories	Thermal insulation performance and material selection	Emphasis on low thermal conductivity and high-temperature stability	Insufficient attention to mechanical property degradation and strengthening strategies	[15]
Reviews on thermal-shock-resistant ceramics	Thermal shock resistance and damage tolerance	Focus on fracture mechanics and thermal stress mitigation	Predominantly centered on dense ceramics; porous systems and their unique challenges are underexplored	[16]
This review	An integrated framework linking degradation mechanisms to multi-scale strengthening strategies for LTIRs	(1) Proposes a three-stage progressive damage model under coupled thermo-mechano-chemical fields; (2) Systematically categorizes strengthening strategies across four dimensions; (3) Analyzes synergistic effects and performance trade-offs; (4) Provides a design map linking service conditions to optimal strategy combinations	/	/

**Table 2:** Recommended characterization methodologies, triggering service conditions, and dominant mechanisms across the three progressive degradation stages of LTIRs.

Degradation Stage	Dominant Mechanism	Key Triggering Service-Condition Parameters	Recommended Characterization Techniques
Stage I: Thermo-mechanically induced microcracking	Thermo-mechanical stress concentration; crack nucleation and propagation at pore boundaries	Temperature: 600~1200 °C Thermal gradient: 50~300 K·cm^−1^ Cycling rate: 5~20 K·min^−1^ Atmosphere: oxidative/neutral, no severe corrosion Time scale: several hundred thermal cycles Load: cyclic thermal stress; low static creep load	In situ high-temperature SEM; X-ray micro CT; digital image correlation (DIC); thermal shock cycling test; post mortem fracture analysis; phase-field modeling; FE simulation of thermal stress distribution
Stage II: Chemical corrosion and oxidation	Alkali/slag corrosion; oxidation of non-oxide phases; formation of low-melting eutectic phases	Temperature: >800 °C Atmosphere: O_2_, steam, CO, and an alkali-vapor/slag-containing environment Time scale: tens to thousands of service hours Load: moderate static thermal–mechanical load	Raman spectroscopy; FTIRs; X-ray micro CT; in situ high-temperature SEM; post-mortem phase and microstructure analysis; corrosion resistance test
Stage III: Creep and grain coarsening	Grain overgrowth; sintering shrinkage; pore coarsening/pore collapse under long-term thermal–mechanical load	Temperature: >1000 °C Load: sustained static creep load Atmosphere: arbitrary (corrosive or relatively clean) Time scale: thousands to tens of thousands of service hours	High-temperature creep testing; X-ray micro CT; post-mortem SEM-EDS; long term isothermal annealing test; pore size distribution measurement

Notes: DIC, digital image correlation; FTIR, Fourier-transform infrared spectroscopy; SEM-EDS, scanning electron microscopy combined with energy-dispersive X-ray spectroscopy.

**Table 3:** Physical mechanisms and performance trade-offs governing isolated, interconnected, and hierarchical multimodal pore architectures in LTIRs.

Pore Architecture Type	Typical PSD Feature	Dominant Mechanical Deformation and Fracture Mode	Governing Thermal Transport Regimes	Service Degradation Resistance and Damage Tolerance
Isolated closed pores	Monomodal (typically 5~50 μm)	Membrane hoop stress distribution; prevents strut buckling; high compressive strength.	Solid conduction restricted to cell struts; suppresses convective transport; ultra-low RT conductivity.	Excellent resistance to stage II alkali/slag penetration; moderate crack-arrest capability.
Interconnected open pores	Broad monomodal or unimodal (1~100 μm)	Asymmetric bending and buckling of cell struts $E/E_{0}\propto {\left(1-P\right)}^{n}$; lower load-bearing capacity.	Significant high-temperature radiative transport; active internal gas convection through open channels.	Poor; accelerates corrosive vapor penetration, deep slag ingress, and creep-induced pore collapse.
Hierarchical multimodal pores	Distinct bimodal/multimodal (50 nm~1 μm paired with 10~60 μm)	Microcrack bifurcation, multi-stage crack deflection, and pore-edge stress dilution; high fracture energy.	Decoupled transport: Knudsen gas-suppression via nanopores + radiative scattering + solid path truncation.	Superior thermal shock damage tolerance (*R⁗*); preserves residual strength and prevents catastrophic spalling.

**Table 4:** Comparison of mechanical properties, thermal performance, and strengthening effectiveness of various strategies for LTIRs.

Strengthening Strategy	Representative Material	Porosity (%)	Pore Size Range (μm)	CCS (MPa)	TC (W/(m·K))	TSR (%)	Key Advantage	Limitation	Ref.
Isolated closed pores	Al_2_O_3_ LTIRs	97.4	40~280	0.76	0.063 (25 °C)	/	Eliminates crack-tip stress concentration	Ineffective at arresting long-range cracks even with reduced corrosion	[39]
Spherical pores	Al_2_O_3_ LTIRs	63.0	/	21.4	0.192 (300 °C)	70% (three cycles, air quenching)	Uniform stress transfer	Complex fabrication; weak interfacial bonding	[47]
Micro–nano pores	Al_2_O_3_ insulating ceramics	82.91	0.1~1; 20~140	14.17	0.0642 (25 °C)	/	Reduces critical defect size; deflects cracks	May compromise TSR with excessive nano-pore fraction, ill-designed hierarchical pore configuration; complex processing	[50]
Hierarchical pores	Mullite thermal insulating ceramics	75.7	<500, mainly <200	26.6	0.279 (25 °C)	/	Bimodal pores synergize crack deflection and phonon scattering	Improper pore-size fractions cause differential sintering and coarsening during prolonged service	[60]
Pore-wall densification	Mullite thermal insulating ceramics	67.7	40~170	41.3	/	61% (three cycles, air quenching)	Enhanced strut strength	Increased density; reduced insulation	[44]
In-site bonded phase	Mullite-bonded thermal insulating ceramics	91.3	10~100	1.22	0.035 (25 °C)	/	Shields tensile stress; dissipates energy	Poor crack bridging; limited large-crack control	[67]
ZrO_2_ particle	Al_2_O_3_-based thermal insulating ceramics	86.7	0.01~150	4.7	0.092 (25 °C)	68.1 (four cycles, water quenching)	Bridging, deflection, pull-out	Agglomeration risk	[70]
Whisker/fiber	Mullite-whisker- reinforced Al_2_O_3_ LTIRs.	74.3	0.1 ~100	45.0	0.189 (25 °C)	/	Relaxes thermal mismatch stress	Difficult thickness-gradient control	[75]
Graded transition layer	Anorthite-based LTIRs	76.1	/	1.84	0.213 (1000 °C)	/	Mitigates abrupt thermal mismatch	Precise gradient control difficult	[81]
Weakly bonded interface	Al_2_O_3_ thermal insulating ceramics	80.2	/	1.7	0.94 (25 °C)	/	Interfacial sliding dissipates energy; enhances damage tolerance	Reduces room-temperature shear strength	[85]
Viscoelastic glassy phase	Anorthite-based LTIRs	62	0~400	6.8	0.109 (25 °C)	/	Viscous stress relaxation at high temperature	Excess glass leads to creep/softening; accelerates corrosion	[89]

Notes: CCS, cold compressive strength; TC, Thermal Conductivity; TSR, Residual Strength Retention; /, data not reported in the referenced study. Representative data are compiled from the cited literature for comparative purposes. All listed data correspond to bulk LTIRs.

## Data Availability

No new data were created or analyzed in this study. Data sharing is not applicable to this article.
